# Ultrasound Characteristics of In Utero Infection

**DOI:** 10.1155/S1064744997000446

**Published:** 1997

**Authors:** Karoline S. Puder, Marjorie C. Treadwell, Bernard Gonik

**Affiliations:** 1 Grace and Hutzel Hospitals Department of Obstetrics and Gynecology Wayne State University Detroit MI USA; 2 Department of Obstetrics and Gynecology Grace Hospital 6071 West Outer Drive Detroit MI 48235 USA

## Abstract

In utero infection of the fetus has become recognized as an important cause of fetal and neonatal morbidity and mortality. Since both anatomic and functional abnormalities have been described in the fetus related to various infections, ultrasonography may be a valuable diagnostic tool in this regard. A complete review of the current literature was undertaken to report available information on this topic. Common pathogens or clinical conditions were selected. The identified data were confounded by the way in which each case originally presented for study. Although certain anomalies were frequently associated with individual organisms, their incidence could not be determined, nor were most specific to that infectious agent. Representative ultrasound images are presented for common and unusual cases.


Infectious Diseases in Obstetrics and Gynecology 5:262–270 (1997)
© 1997 Wiley-Liss, Inc.

### Ultrasound Characteristics of In Utero Infection

**Karoline S. Puder,* Marjorie C. Treadwell, and Bernard Gonik**
*Grace and Hutzel Hospitals, Department of Obstetrics and Gynecology, Wayne State University, Detroit, MI*

ABSTRACT

**In utero infection of the fetus has become recognized as an important cause of fetal and neonatal morbidity and mortality. Since both anatomic and functional abnormalities have been described in the fetus related to various infections, ultrasonography may be a valuable diagnostic tool in this regard. A complete review of the current literature was undertaken to report available information on this topic. Common pathogens or clinical conditions were selected. The identified data were confounded by the way in which each case originally presented for study. Although certain anomalies were frequently associated with individual organisms, their incidence could not be determined, nor were most specific to that infectious agent. Representative ultrasound images are presented for common and unusual cases. Infect. Dis. Obstet. Gynecol. 5:262–270, 1997.** © 1997 Wiley-Liss, Inc.

KEY WORDS
congenital anomalies; teratogenesis; ultrasonography

In utero infection of the fetus has become recognized as an important cause of fetal and neonatal morbidity and mortality. As our awareness of the number of pathogenic organisms increases, so too does our appreciation of the potential for in utero diagnosis of these infections. Both anatomic and functional abnormalities in the fetus have been described for many types of infection. The focus of this report is on the use of ultrasonography as a tool to recognize, and monitor, these infectious complications. Although a great deal of recent literature has become available concerning invasive means (e.g., amniocentesis, cordocentesis) to diagnose in utero infection, this is beyond the scope of the current review.

Most of the data to be presented are derived from limited sources, including case reports or small case series. These sources are biased by the way the case presents, i.e., if infection was recognized prospectively, or if a given anomaly led to a thorough workup of the patient which included potential infectious agents. Therefore, associations may be drawn, but absolute cause and effect relationships should be tempered by the quality of the data. Similarly, an accurate incidence of a given ultrasound-based anomaly is hard to establish due to the rarity of most infectious processes.

### VIRAL INFECTIONS

### Cytomegalovirus (CMV)

CMV remains the most common congenital virus infection, affecting an estimated 0.4–2.3% of all liveborn infants.[1,2] The virus itself is a double-stranded DNA herpes virus with multiple strains identified. An increased incidence of seropositivity is encountered in lower socioeconomic strata with variation between regions of the country. Diagnosis of a primary maternal infection requires demonstration of the presence of CMV-IgM antibodies in the absence of CMV-IgG, as well as seroconversion of CMV-specific IgG antibody from negative to positive. Presence of IgG antibodies without documented seroconversion is not helpful in diagnosing the primary maternal infection thought responsible

*Correspondence to: Dr. Karoline S. Puder, Department of Obstetrics and Gynecology, Grace Hospital, 6071 West Outer Drive, Detroit, MI 48235.

Received 3 September 1996

Accepted 23 June 1997

Review Article

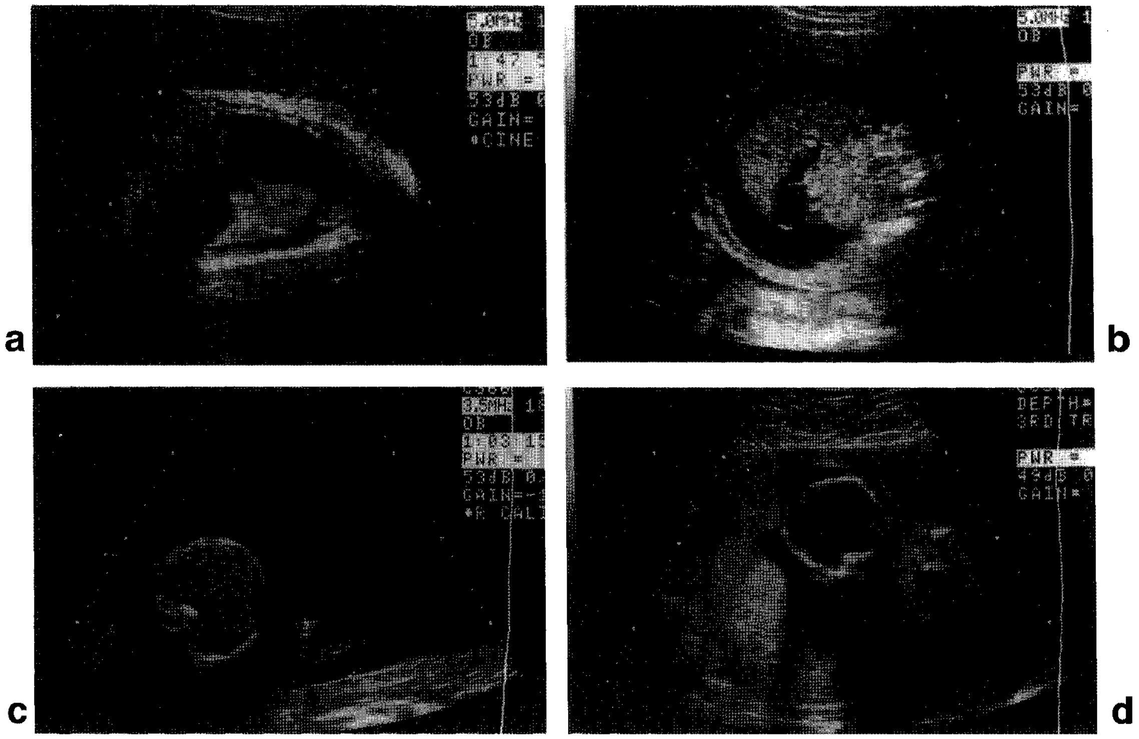

Fig. 1. Ultrasound manifestations of non-immune fetal hydrops: (a) hydrothorax, (b) ascites, (c) polyhydramnios, (d) scalp edema.

for in utero infection. Approximately 2% of seronegative patients will convert during pregnancy. Of these primary CMV infections, 40% result in fetal infection. Only 10% of fetal infections result in significant disease.[3] It is in this group that ultrasound findings consistent with fetal infection would be expected. The role of prenatal diagnosis of fetal infection is not clear in the absence of ultrasound evidence of fetal disease. Isolation of CMV from amniotic fluid was first reported in 1971,[4] and amniotic fluid viral culture remains the gold standard for diagnosis of fetal infection. The use of fetal blood sampling for culture and detection of CMV IgM is not as sensitive as amniocentesis.[5] Non-specific serologic findings including anemia, thrombocytopenia, elevated liver function tests, and elevated total IgM levels support a diagnosis of in utero infection but do not necessarily correlate with severity of fetal disease.

Many of the manifestations of CMV infection in the neonate may be detected prenatally and warrant further evaluation. The ultrasound findings associated with CMV are non-specific and include hepatosplenomegaly (74%), microcephaly (50%), intrauterine growth retardation (IUGR) (41%), and cerebral calcifications.[6] The presence of fetal ascites and/or non-immune hydrops (Fig. 1) is more frequently noted with severe infections. The intracranial calcifications may be accompanied by ventriculomegaly (Fig. 2) or intrahepatic calcifications, however, mild ventriculomegaly may be an isolated finding. Subtle signs include oligo- or polyhydramnios. Less common ultrasound findings described include pulmonary hypoplasia, isolated pericardial or pleural effusions, and echogenic bowel (Fig. 3).[7,8] Discordant effects have been described in multiple gestations.[9,10]

As with all fetal infections, the ultrasound diagnosis of abnormalities indicates a more severe disease and worse prognosis. The findings associated with CMV are not unique to this infection, however, the relative frequency of CMV infection makes it more commonly encountered. Any patient with ultrasound findings of in utero infection warrants inclusion of CMV in the differential.

### Rubella

Rubella (German measles) is a well-characterized, mild exanthematous disease of childhood caused by a single-stranded RNA virus of the togavirus

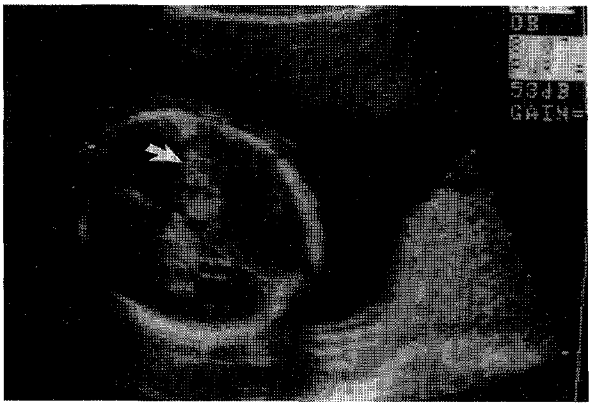

Fig. 2. Ventricular dilatation with scattered calcifications (arrow), consistent with CMV infection.

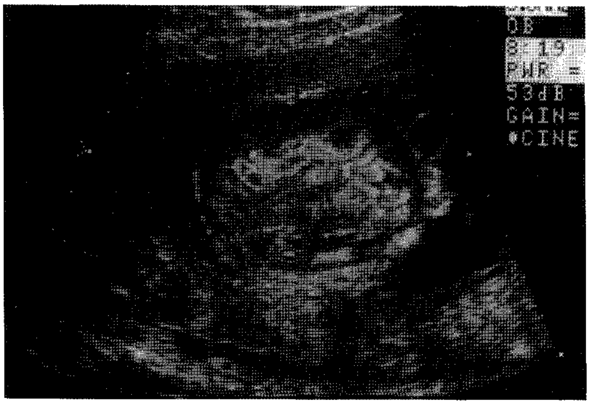

Fig. 3. Echogenic bowel; note loops of small intestine outlined by calcifications.

family. In the pregnant host, this disease can have catastrophic effects on the fetus, as first described by Gregg in 1941.[11] The last epidemic in the prevaccine era (1964–1965) resulted in approximately 11,000 miscarriages, abortions, and stillbirths, and approximately 20,000 cases of congenital rubella syndrome in the newborn.[12] Since the introduction of an effective vaccine in 1969, the incidence of clinical infection has dramatically declined, although there has been a recent resurgence of rubella identified by the Centers for Disease Control and Prevention (CDC).[13]

Based on reported *neonatal* clinical characteristics of the disease, ultrasound-identified manifestations of *in utero* infection could include cardiovascular defects (pulmonary artery hypoplasia, coarctation of the aorta), microphthalmos, microcephaly, polycystic kidney, IUGR, and hepatosplenomegaly. A less likely identifiable abnormality, yet relatively common finding in the infected newborn, is

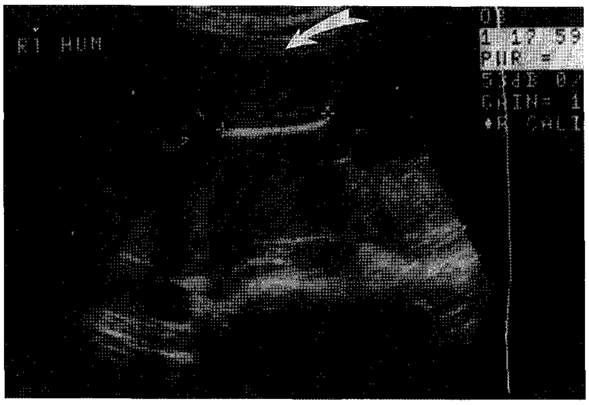

Fig. 4. Large septated desquamating skin lesion (arrow) of right upper extremity in congenital HSV infection.

congenital cataracts. Other typical features of congenital rubella infection, including hearing loss, purpura, and mental retardation, cannot be identified by ultrasound imaging. It is interesting to note that due to the difference in time frames between the peak occurrence of this congenital infectious disease and the availability of ultrasonography, no reported ultrasound-based cases have ever been published in the literature.

### Herpes Simplex Virus (HSV)

Congenital HSV infection is often severe, with a high morbidity and mortality. Most cases are acquired during delivery through an infected birth canal. Intrauterine infection may also occur, either through transplacental passage of virus or ascending local infection. Transplacental infection may result in a neonate born with skin lesions, multisystem organ failure, and central nervous system (CNS) lesions consistent with destruction of developing brain tissue (microcephaly, microphthalmia, chorioretinitis, hydranencephaly, multicystic encephalomalacia). Several cases of prenatally detected lesions of congenital HSV infection have been reported. Reports of destructive intracranial lesions include hydranencephaly diagnosed on a 30 week ultrasound[14] and ventriculomegaly, enlarged cisterna magna, and 4th ventricle cyst on a 30 week ultrasound, in the presence of a normal 22 week study.[15] Non-immune hydrops, with skin edema, pleural effusion, and ascites, has also been reported as a manifestation of this congenital infection.[16] A recent report detected large desquamating skin lesions (Fig. 4), restricted fetal movement, lymphadenopathy (Fig. 5), and intrahepatic calcifications

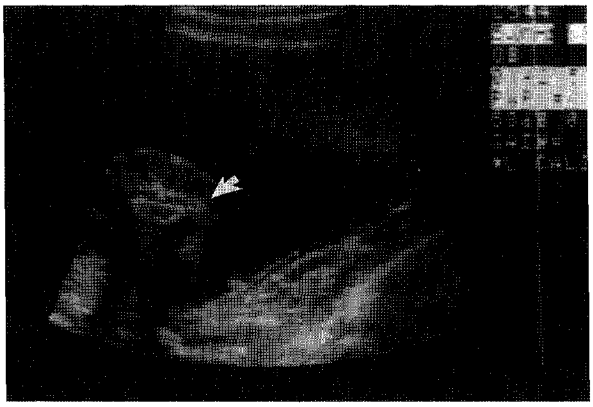

Fig. 5. Multiple echogenic masses adjacent to the cervical spine (arrow), consistent with cervical lymphadenopathy.

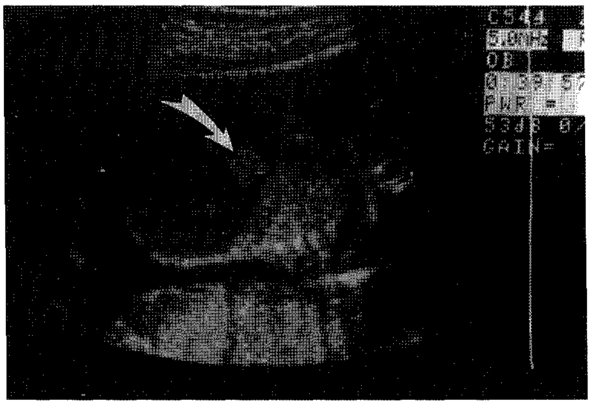

Fig. 6. Coronal view of the fetal thorax and abdomen; note the large areas of calcification within the fetal liver (arrow).

(Fig. 6) on an ultrasound performed at 19 weeks.[17] The correct diagnosis was confirmed with invasive testing and supported by studies after termination of the pregnancy. The destructive CNS lesions tend to be more severe than those seen with other infections. The bullous skin lesions appear unique to HSV.

### Human Immunodeficiency Virus (HIV)

Infection with HIV leads to a chronic, progressive illness culminating in the acquired immunodeficiency syndrome (AIDS) and death. The shifting epidemiology of this infectious agent is highlighted by the fact that women of reproductive age are now one of the most rapidly growing populations with HIV infection. In pregnancy, evidence of maternal to fetal transmission is well documented. This can occur early in gestation or during the labor and delivery process. Marion et al.[18,19] were the first to describe a dysmorphic syndrome in children exposed to HIV in utero. Features of this embryopathy included growth failure (75%), microcephaly (70%), and craniofacial abnormalities consisting of ocular hypertelorism (50%), prominent forehead (75%), flat nasal bridge (70%), obliquity of the eyes (65%), long palpebral fissures (60%), short nose (65%), and patulous lips (60%). The authors postulated that the variable expression of these HIV-associated physical findings may relate to the timing of exposure in pregnancy. It is now believed that the above findings are not specific to HIV, but are secondary to confounding factors, such as ethnicity, poor nutrition, substance abuse, and concomitant infections. Ades et al.,[20] from the European Collaborative Study, did not detect 1 case of HIV dysmorphic syndrome in more than 600 cases studied. To date, no ultrasound-based data exist describing the in utero identification of the HIV-infected fetus.

### Varicella-Zoster Virus (VZV)

Maternal VZV infection had been identified to result in transplacental infection of the fetus. The clinical spectrum of disease varies from asymptomatic seroconversion in the neonate to severe forms of symptomatic congenital VZV syndrome. This latter condition has been identified in utero by ultrasonography and can be categorized into two types: a virus-specific deformation sequence and a non-specific, non-immune hydrops-like picture. The deformation sequence consists of some combination of the following ultrasound findings: limb hypoplasia, cicatricial skin lesions, microphthalmos, and abnormal positioning of extremities. Focal intracranial calcifications, club feet, polyhydramnios, hydrocephalus, liver echogenicities, and arthrogryposis, although not specific for VZV infection, have also been reported.[21] Balducci et al.,[22] however, prospectively examined pregnant women with VZV infection throughout gestation and noted that in utero ultrasound detection of structural abnormalities associated with VZV infection was rare. In their study, 36 women with first trimester chickenpox were examined with ultrasonography at 16–20 weeks of gestation with no abnormalities identified in any of the fetuses. Scattered case reports do present convincing evidence for in utero infection. For example, Scharf et al.[23] documented maternal rash, positive neonatal virologic studies for

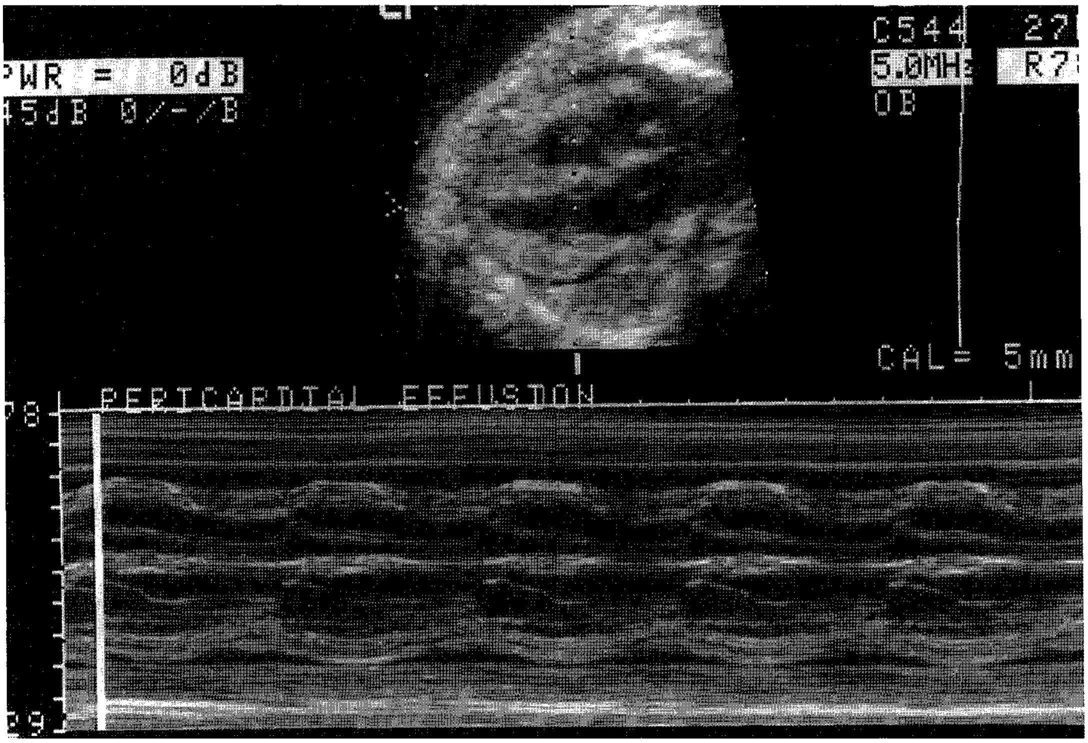

Fig. 7. Pericardial effusion shown in both real time and M-mode ultrasound.

VZV and antenatal ultrasound findings of massive hydramnios, bilateral hydrocephalus, bilateral malpositioning of hands and feet, and skin edema in one case.

These findings are tempered by others noting that these lesions develop over time and fetuses at risk should be followed serially. Several authors have cautioned that too early an examination of the fetus may miss evidence of fetal infection. Pretorius et al.[21] performed serial ultrasound examinations in VZV-infected gravidas and noted a latency period of at least 5 weeks before ultrasound findings of infection were detected. Similarly, Harding and Baumer[24] reported on a case of maternal varicella infection at 14 weeks gestation with a normal ultrasound examination at 17 weeks, and a severely compromised neonate with contractures and postural deformities involving all extremities at delivery. Additionally, a report of maternal varicella at 11 weeks gestation, with serial ultrasonography every 4 weeks, did not reveal abnormalities until 22 weeks, when atrophy of the muscles of the left leg and malposition of the left foot were noted.[25]

The second type of in utero presentation, nonimmune hydrops, also appears long after the initial maternal VZV infection has subsided. Three gravidas with varicella infection before 20 weeks of gestation were noted to have ultrasound features in the fetus consistent with viral infection only in the third trimester.[26–28] These findings included hepatomegaly, ascites, pleural effusion, pericardial effusion, and liver calcifications.

### Parvovirus

Parvovirus B19 may infect both children and adults, and generally has a benign course in these patients. Significant disease in the fetus may be caused by in utero infection. Sonographic signs of fetal infection have been reported from 3 to 12 weeks after maternal infection.[29] Numerous case reports have documented a broad range of antenatal ultrasound findings in infected fetuses.[30–40] When abnormalities are detected during antenatal ultrasound, they are almost exclusively abnormal fluid collections and are believed to be due to fetal anemia and/or myocarditis. Therefore, placental thickening, polyhydramnios, pleural and pericardial effusions (Fig. 7), ascites, skin edema, and cardiomegaly are the most frequently found manifestations.

Others cases may present as a fetal demise,[35] twin gestation with clinical findings of twin-twin transfusion syndrome,[36] or oligohydramnios.[37] Congenital anomalies have rarely been associated with documented parvovirus infection. Although anomalies are suggested by a report of a fetus diagnosed at 13 weeks (cystic hygroma and no midline intracranial echo) with progression at 16 weeks (no midline intracranial echo, thick nuchal fold, fetal hands and feet in abnormally flexed position, growth at less than the 5th percentile), these may be coincidental. Despite a normal karyotype, the pregnancy was terminated and parvovirus was found in multiple tissues, though none was found in maternal blood.[38] A recent report of two cases of hydrocephalus in association with this fetal infection, after normal second trimester ultrasound examinations,[40] does raise the possibility that congenital anomalies may be in the spectrum of disease.

The in utero natural history of this disease process, in particular the ultrasound-related findings, is poorly defined. This is due to the relative rarity of this condition, the variable gestational age at diagnosis, and the now common attempt at therapeutic intervention (e.g., in utero transfusion). However, serial ultrasound studies of even severely affected fetuses have shown resolution of the hydropic changes.[33] Humphrey and colleagues[39] reported on a 26 week fetus with parvovirus infection and hydrops, anhydramnios, thickened placenta, bilateral pleural effusions, cardiomegaly, pericardial effusion, and marked ascites. With observation, these findings had all resolved by 32 weeks gestation.

### BACTERIAL INFECTIONS

### Syphilis

The incidence of syphilis in the general U.S. population has increased significantly over the past few years, with a current rate of approximately 14–15 cases per 100,000 persons.[41] Although the exact incidence in pregnancy is undetermined, a 4-fold increase in congenital syphilis cases has recently been reported.[42] Unfortunately, congenital syphilis is first suspected in many cases when the ultrasound report demonstrates a fetal demise,[43] consistent with the 22% incidence of fetal demise in mothers with syphilis reported in the 1930s.[42] These features of fetal demise have been well described elsewhere.[44] Fetal infection can be diagnosed in utero, and may be the cause of ultrasonographic findings of non-immune hydrops. Classically, the feature of placental thickening (Fig. 8) is reported in the literature as consistent with this in utero infection. Additional findings may include ascites and skin edema.[45,46] Although the data are limited, Nathan et al.[47] reported the incidence of ultrasound-detected hepatomegaly to be 57%, placental thickening 62%, and ascites 17% in infected fetuses. Ultrasound detection of dilatation of fetal bowel has been reported and is associated with fibrotic and infiltrative processes causing obstruction.[48,49] The characteristic bone findings of congenital syphilis have also been detected in utero.[50] Both abnormal bone morphometry (three standard deviations below normal for gestational age) and morphology (thickening, curvature, and multiple areas of bowing) were described. Despite the numerous other potential observable ultrasound features of congenital syphilis, none has been reported in the recent literature. Data are lacking with regard to the effect of aggressive antimicrobial therapy and the resolution of syphilis-related ultrasonographic abnormalities.

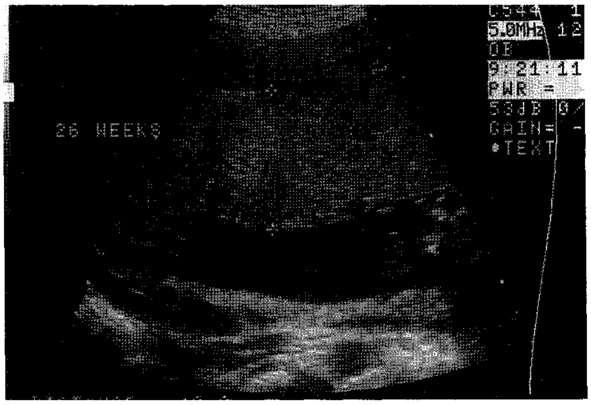

Fig. 8. Isolated placental thickening (>4 cm) in a 26 week gestation consistent with in utero syphilis infection.

### Intraamniotic Infection (Chorioamnionitis)

As previously mentioned, in addition to anatomic abnormalities, ultrasound-based functional aberrations in the fetus have been reported which are associated with infection. Vintzileos and colleagues[51] were the first to introduce the concept of using biophysical profile testing to detect occult fetal infection. In their study of 73 gravidas with premature rupture of the membranes, a low biophysical score ($\leq 7$) was a good predictor of impending fetal infection. When subdivided into individual components, the first manifestations of impending infection were loss of fetal breathing and non-reactive non-stress testing. Conversely, the presence of fetal breathing had the highest specificity in predicting absence of fetal infection. In this series, testing was done on a daily basis, and it appears that this frequency is necessary to provide accurate fetal assessment related to infection.

Use of biophysical profile testing for subclinical infection remains controversial. Miller et al.[52] were not able to identify a relationship between any component of the biophysical profile and subsequent clinical chorioamnionitis. This study was limited by the absence of any identified neonatal sepsis in the study group. This has prompted the suggestion that biophysical profile testing may be more accurate in its ability to detect impending subclinical fetal infection as opposed to maternal infection. The rationalization for this latter perspective is that the loss of reactivity, movement, breathing, and tone in the fetus is similar to these same non-specific signs seen with newborn sepsis in the nursery. In a recent study by Carroll et al.,[53] study subjects with premature rupture of the membranes underwent ultrasound testing, cordocentesis, and amniotic fluid sampling in an attempt to identify in utero infection. Although there was a tendency for lower biophysical profile scores and amniotic fluid indices, the majority of subjects with documented in utero infection had normal ultrasound-based testing.

### PARASITIC INFECTIONS

### Toxoplasma

Antenatal infection with *Toxoplasma gondii* is seen frequently in Europe and may cause significant sequelae. The initial infection leads to focal tissue necrosis and destruction, most often in the brain, heart, lungs, and liver. Classic manifestations of severe disease include chorioretinitis, convulsions, hydrocephalus, and intracranial calcifications. Of these, the latter two may be diagnosed by antenatal ultrasound.

Abnormalities of the cerebral ventricles (Fig. 9), ranging from mild ventriculomegaly to severe hydrocephalus,[54] and in rare instances to hydranencephaly,[55] are the most frequent prenatal ultrasound findings in this infection. The dilatation of the ventricles is usually symmetrical[56] and may develop weeks after the prenatal diagnosis has been made.[57–59]

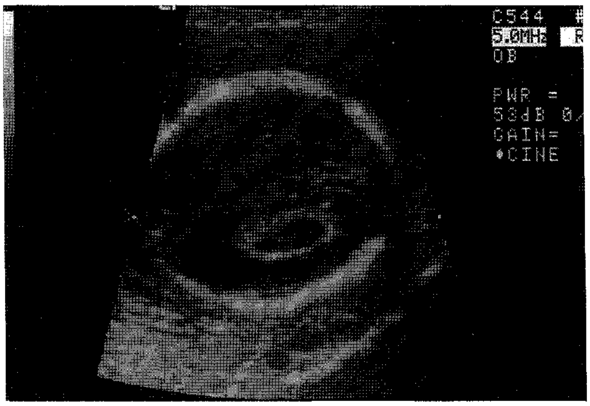

Fig. 9. Periventricular calcifications.

Intracranial calcifications without distinct pattern may be seen in utero.[56] Characteristic intracranial findings and their progression have also been noted in utero, with documented fetal toxoplasmosis infection.[60] Mild ventriculomegaly and periventricular hyperechoic areas of $\leq 2$ mm were first detected at 30 weeks of gestation and repeat ultrasound at 33 weeks revealed progressive hydrocephalus, increase in the size (10 mm) and number of hyperechoic areas, and polyhydramnios. Non-specific findings of infection have also been documented: ascites, hepatomegaly, increased placental thickness, calcifications in the fetal liver, pericardial and pleural effusions, oligohydramnios, and intrauterine growth restriction.[56,61–63] Though the presence of ultrasound findings may prompt investigation, it should be noted that the majority (approximately 80%) of prenatally diagnosed cases of congenital toxoplasmosis do not have detectable ultrasound abnormalities.[54,59,61,64]

### CONCLUSIONS

Although some infections acquired in utero have classic ultrasound findings, there is a significant overlap between infectious agents. Non-immune hydrops, intrauterine growth restriction, ventriculomegaly, and fluid abnormalities form a significant component of the ultrasound findings in these infections. Therefore, in the absence of more specific features, any patient warranting evaluation for these findings would benefit from the inclusion of infectious etiologies in the diagnostic process.

### REFERENCES

1. Stagno S, Pass RF, Alford CA: Perinatal infections and maldevelopment. In Bloom AD, James LS (eds): The

Fetus and the Newborn. New York: Alan R. Liss, 31–50, 1981.
2. Demmler GJ: Summary of a workshop on surveillance for congenital CMV. Rev Infect Dis 13:315–329, 1991.
3. Adler SP: Cytomegalovirus and pregnancy. Curr Opin Obstet Gynecol 4:670–675, 1992.
4. Davis LE, Tweed GV, Chin TDY, Miller GL: Intrauterine diagnosis of cytomegalovirus infection: Viral recovery from amniocentesis fluid. Am J Obstet Gynecol 109:1214–1219, 1971.
5. Dinner C, Leisnard C, Content J, Busine A, Aderca J, Rodesch F: Prenatal diagnosis of 52 pregnancies at risk for congenital CMV infection. Obstet Gynecol 82:481–486, 1993.
6. Guerina NG: Management strategies for infectious disease in pregnancy. Semin Perinatol 18:305–320, 1994.
7. Lynch L, Daffos F, Emanuel D, et al.: Prenatal diagnosis of fetal CMV infection. Am J Obstet Gynecol 165: 714–718, 1991.
8. Edelman KA, Levine AB, Chitkara U, Berkowitz RL: Reliability of pleural fluid lymphocyte counts in the antenatal diagnosis of congenital chylothorax. Obstet Gynecol 78:530–532, 1991.
9. Hohlfeld P, Vial Y, Maillard-Brignon C, Vaudaux B, Fawer CL: Cytomegalovirus fetal infection: Prenatal diagnosis. Obstet Gynecol 78:615, 1991.
10. Schneeberger PM, Groenendaal F, DeVries LS, Van Loon AM, Vroom TM: Variable outcome of a congenital CMV infection in a quadruplet after a primary infection of the mother during pregnancy. Acta Paediatr 83:986–989, 1994.
11. Gregg NM: Congenital cataract following German measles in the mother. Trans Ophthalmol Soc Aust 3: 35, 1941.
12. Oshiro BT, Monga M, Graham JM: Mumps, measles, rubella, and roseola. In Gonik B (ed): Viral Diseases in Pregnancy. New York: Springer-Verlag, pp 236–247, 1994.
13. Centers for Disease Control and Prevention: Increase in rubella and congenital rubella syndrome—United States, 1988–1990. MMWR 40:93–98, 1991.
14. Parish WR: Intrauterine herpes simplex virus infection: Hydranencephaly and a nonvesicular rash in an infant. Int J Dermatol 28:397–401, 1989.
15. Sarkell B, Blaylock WK, Vernon H: Congenital neonatal herpes simplex virus infection. J Am Acad Dermatol 27:817–821, 1992.
16. Greene D, Watson WJ, Wirtz PS: Non-immune hydrops associated with congenital herpes simplex infection. SD J Med 46:219–220, 1993.
17. Lanouette MJ, Duquette DA, Jacques SM, Quershi F, Johnson MP, Berry SM: Prenatal diagnosis of fetal herpes simplex infection. Fetal Diagn Ther 11:414–416, 1996.
18. Marion RW, Wiznia AA, Hutcheon RG, Rubinstein A: Human T-cell lymphotropic virus type III (HTLV-III) embryopathy. A new dysmorphic syndrome associated with intrauterine HTLV-III infection. Am J Dis Child 140:638–640, 1986.
19. Marion RW, Wiznia AA, Hutcheon RG, Rubinstein A: Fetal AIDS syndrome score. Correlation between severity of dysmorphism and age at diagnosis of immunodeficiency. Am J Dis Child 141:429–431, 1987.
20. Ades AE, Parker S, Berry T, et al.: Prevalence of maternal HIV-1 infection in Thames regions: Results from anonymous unlinked neonatal testing. Lancet 337: 1562–1565, 1991.
21. Pretorius DH, Hayward I, Jones KL, Stamm E: Sonographic evaluation of pregnancies with maternal varicella infection. J Ultrasound Med 11:459–463, 1992.
22. Balducci J, Rodis JF, Rosengren S, Vintzileos AM, Spivey G, Vosseller C: Pregnancy outcome following first-trimester varicella infection. Obstet Gynecol 79:5–6, 1992.
23. Scharf A, Scherr O, Enders G, Helftenbein E: Virus detection in the fetal tissue of a premature delivery with a congenital varicella syndrome. A case report. J Perinat Med 18:317–322, 1990.
24. Harding B, Baumer JA: Congenital varicella-zoster. A serologically proven case with necrotizing encephalitis and malformation. Acta Neuropathol 76:311–315, 1988.
25. Pons JC, Rozenberg F, Imbert MC, et al.: Prenatal diagnosis of second-trimester congenital varicella syndrome (letter). Prenat Diagn 12:975–976, 1992.
26. Vincent Y, Bernard JP, Laroussinie MP, Lafay Pillet MC, Taurelle R: Varicelle et grossesse. Rev Fr Gynecol Obstet 87:490–492, 1992.
27. Lecuru F, Taurelle R, Bernard JP, et al.: Varicella zoster virus infection during pregnancy: The limits of prenatal diagnosis. Eur J Obstet Gynecol Reprod Biol 56:67–68, 1994.
28. Cuthbertson G, Weiner CP, Giller RH, Grose C: Prenatal diagnosis of second-trimester congenital varicella syndrome by virus-specific immunoglobulin M. J Pediatr 111:592–595, 1988.
29. Rodis JF, Vintzileos AM: Parvovirus. In Gonik B (ed): Viral Diseases in Pregnancy. New York: Springer-Verlag, pp 196–214, 1994.
30. Maeda H, Shimokawa H, Satoh S, Nakano H, Nunoue T: Nonimmunologic hydrops fetalis resulting from intrauterine human parvovirus B-19 infection: Report of two cases. Obstet Gynecol 72:482–485, 1988.
31. Anand A, Gray ES, Brown T, Clewley JP, Cohen BJ: Human parvovirus infection in pregnancy and hydrops fetalis. N Engl J Med 316:183–186, 1987.
32. Peters MT, Nicolaides KH: Cordocentesis for the diagnosis and treatment of human fetal parvovirus infection. Obstet Gynecol 75:501–504, 1990.
33. Pryde PG, Nugent CE, Pridjian G, Barr M Jr, Faix RG: Spontaneous resolution of nonimmune hydrops fetalis secondary to human parvovirus B19 infection. Obstet Gynecol 79:859–861, 1992.
34. Pustilnik TB, Cohen AW: Parvovirus B19 infection in a twin pregnancy. Obstet Gynecol 83:834–836, 1994.
35. Morey AL, Keeling JW, Porter HJ, Fleming KA: Clinical and histopathological features of parvovirus B19 infection in the human fetus. Br J Obstet Gynaecol 99: 566–574, 1992.

36. Weiner CP, Naides SJ: Fetal survival after human parvovirus B19 infection: Spectrum of intrauterine response in a twin gestation. Am J Perinatol 9:66–68, 1992.
37. Panero C, Azzi A, Carbone C, Pezzati M, Mainardi G, diLollo S: Fetoneonatal hydrops from human parvovirus B19. Case report. J Perinat Med 22:257–264, 1994.
38. Tiessen RG, Van Elsacker-Niele AMW, Vermeij-Keers CHR, Oepkes D, Van Roosmalen J, Gorsira MCB: A fetus with a parvovirus B19 infection and congenital anomalies. Prenat Diagn 14:173–176, 1994.
39. Humphrey W, Magoon M, O'Shaughnessy R: Severe nonimmune hydrops secondary to parvovirus B-19 infection: Spontaneous reversal in utero and survival of a term infant. Obstet Gynecol 78:900–902, 1991.
40. Katz VL, McCoy MC, Kuller JA, Hansen WF: An association between fetal parvovirus B19 infection and fetal anomalies: A report of two cases Am J Perinatol 13:43–45, 1996.
41. Hanlon-Lundberg KM, Ismail MA: Syphilis. In Pastorek JG (ed): Obstetric and Gynecologic Infectious Disease. New York: Raven Press, pp 479–490, 1994.
42. McIntosh K: Congenital syphilis—Breaking through the safety net. N Eng J Med 323:1339–1341, 1990.
43. Wendel GD, Maberry MC, Christmas JT, Goldberg MS, Norgard MV: Examination of amniotic fluid in diagnosing congenital syphilis with fetal death. Obstet Gynecol 74:967, 1989.
44. Seeds JW, Chescheir NC: Fetal death. In Chervenak FA, Isaacson GC, Campbell S (eds): Ultrasound in Obstetrics and Gynecology. Boston: Little, Brown, pp 1577–1582, 1993.
45. Wendel GD Jr, Sanchez PJ, Peters MT, Harstad TW, Potter LL, Norgard MV: Identification of treponema pallidum in amniotic fluid and fetal blood from pregnancies complicated by congenital syphilis. Obstet Gynecol 78:890–894, 1991.
46. Hallak M, Peipert JF, Ludomirsky A, Byers J: Nonimmune hydrops fetalis and fetal congenital syphilis. J Reprod Med 37:173–176, 1992.
47. Nathan L, Twickler DM, Peters MT, Sanchez PJ, Wendel GD Jr: Fetal syphilis: Correlation of sonographic findings and rabbit infectivity testing of amniotic fluid. J Ultrasound Med 2:97–101, 1993.
48. Satin AJ, Twickler DM, Wendel GD Jr: Congenital syphilis associated with dilation of fetal small bowel. J Ultrasound Med 11:49–52, 1992.
49. Hill LM, Maloney JB: An unusual constellation of sonographic findings associated with congenital syphilis. Obstet Gynecol 78:895, 1991.
50. Raafat NA, Birch AA, Altieri LA, Felker RE, Smith WC, Emerson DS: Sonographic osseous manifestations of fetal syphilis: A case report. J Ultrasound Med 12:783–785, 1992.
51. Vintzileos AM, Campbell WA, Nochimson DJ, Connolly ME, Fuenfer MM, Hoehn GJ: The fetal biophysical profile in patients with premature rupture of the membranes—An early predictor of fetal infection. Am J Obstet Gynecol 152:510–516, 1985.
52. Miller JM Jr, Kho MS, Brown HL, Gabert HA: Clinical chorioamnionitis is not predicted by an ultrasonic biophysical profile in patients with premature rupture of membranes. Obstet Gynecol 76:1051–1054, 1990.
53. Carroll SG, Papaioannou S, Nicolaides KH: Assessment of fetal activity and amniotic fluid volume in the prediction of intrauterine infection in preterm prelabor amniorrhexis. Am J Obstet Gynecol 172:1427–1435, 1995.
54. Desmonts G, Forestier F, Thulliez PH, Daffos F, Capella-Pavlovsky M, Chartier M: Prenatal diagnosis of congenital toxoplasmosis. Lancet 1:500–504, 1985.
55. Bambirra EA, Pittella JE, Rezende M: Toxoplasmosis and hydranencephaly. N Engl J Med 306:1112–1113, 1982.
56. Stray-Pedersen B: Toxoplasmosis in pregnancy. Bailliere's Clin Obstet Gynecol 7:107–137, 1993.
57. Matsui D: Prevention, diagnosis, and treatment of fetal toxoplasmosis. Clin Perinatol 21:675–689, 1994.
58. Hohlfeld P, Daffos F, Thulliez P, et al.: Fetal toxoplasmosis: Outcome of pregnancy and infant follow-up after in utero treatment. J Pediatr 115:765–769, 1989.
59. Daffos F, Forestier F, Capella-Pavlovsky M, et al.: Prenatal management of 746 pregnancies at risk for congenital toxoplasmosis. N Engl J Med 318:271–275, 1988.
60. Desai MB, Kurtz AB, Martin ME, Wapner RJ: Characteristic findings of toxoplasmosis in utero: A case report. J Ultrasound Med 13:60–62, 1994.
61. Pratlong F, Boulot P, Issert E, et al.: Fetal diagnosis of toxoplasmosis in 190 women infected during pregnancy. Prenat Diagn 14:191–198, 1994.
62. Favre R, Grange G, Gasser B: Congenital toxoplasmosis in twins: A case report. Fetal Diagn Ther 9:264–268, 1994.
63. Gross SJ, Phillips OP, Shulman LP, et al.: Adverse perinatal outcome in patients screen-positive for neural tube defects and fetal Down syndrome. Prenat Diagn 14:609–613, 1994.
64. Foulon W, Naessens A, Mahler T, de Waele M, de Catte L, de Meuter F: Prenatal diagnosis of congenital toxoplasmosis. Obstet Gynecol 76:769–772, 1990.
